# Multidrug-resistant bacteria with ESBL genes: a growing threat among people living with HIV/AIDS in Nepal

**DOI:** 10.1186/s12879-022-07503-2

**Published:** 2022-06-07

**Authors:** Riju Maharjan, Anup Bastola, Nabaraj Adhikari, Komal Raj Rijal, Megha Raj Banjara, Prakash Ghimire, Upendra Thapa Shrestha

**Affiliations:** 1 Central Department of Microbiology, Tribhuvan University, Kirtipur, Kathmandu, Nepal; 2 Sukraraj Tropical and Infectious Disease Hospital, Teku, Kathmandu, Nepal

**Keywords:** PLHA, Lower respiratory tract infection, ESBL, *Bla*_CTX-M_, *Bla*_TEM_

## Abstract

**Background:**

Bacterial opportunistic infections are common in people living with HIV/AIDS (PLHA). Besides HIV-TB co-infection, lower respiratory tract infections (LRTIs) due to multidrug-resistant (MDR) bacteria cause significant morbidity and mortality among PLHA. This study identified bacterial co-infection of the lower respiratory tract and detected plasmid-mediated *bla*_TEM_ and *bla*_CTX-M_ genes among Extended-Spectrum β-Lactamase (ESBL) producing isolates from sputum samples in PLHA.

**Methods:**

A total of 263 PLHA with LRTIs were enrolled in this study, out of which, 50 were smokers, 70 had previous pulmonary tuberculosis, and 21 had CD4 count < 200 cells/µl. Sputum samples collected from PLHA were processed with standard microbiological methods to identify the possible bacterial pathogens. The identified bacterial isolates were assessed for antibiotic susceptibility pattern using modified Kirby Bauer disk diffusion method following Clinical Laboratory Standard Institute (CLSI) guidelines. In addition, plasmid DNA was extracted from MDR and ESBL producers for screening of ESBL genes; *bla*_CTX-M_ and *bla*_TEM_ by conventional PCR method using specific primers.

**Results:**

Of 263 sputum samples, 67 (25.48%) showed bacterial growth. Among different bacterial pathogens, *Klebsiella pneumoniae,* (17; 25.37%) was the most predominant, followed by *Haemophillus influenzae,* (14; 20.90%) and *Escherichia coli*, (12; 17.91%). A higher infection rate (4/8; 50%) was observed among people aged 61–70 years, whereas no infection was observed below 20 years. About 30.0% (15/50) of smokers, 32.86% (23/70) cases with previous pulmonary tuberculosis, and 52.38% (11/21) with CD4 count < 200 cells/µl had bacterial LRTIs. Among 53 bacterial isolates excluding *H. influenzae*, 28 isolates were MDR and 23 were ESBL producers. All ESBL producers were sensitive to colistin and polymyxin B. Among ESBL producers, 47.83% (11/23) possessed *bla*_CTX-M_, 8.6% (2/23) were positive for *bla*_TEM_ gene, and 43.48% (10/23) possessed both ESBL genes.

**Conclusion:**

The increasing rate of MDR bacterial infections, mainly ESBL producers of LRTIs causes difficulty in disease management, leading to high morbidity and mortality of PLHA. Hence, it is crucial to know the antibiogram pattern of the isolates to recommend effective antimicrobial therapy to treat LRTIs in PLHA.

## Background

Human Immunodeficiency Virus (HIV), first confirmed in 1981, has killed more than one million people worldwide. Thirty-eight million people were living with HIV/AIDS (PLHA) at the end of 2019, with 1.8 million people becoming newly infected [1]. There are 37,596 PLHA in Nepal, with 2416 new cases in 2020. Unsafe sexual behavior among the heterosexual population is the main route of HIV transmission, followed by unsafe injecting behavior in the Nepalese context [2]. Once, the virus penetrates the host, it destroys the function of immune cells like helper T cells specifically CD4 + T cells, macrophages, and dendritic cells, reducing the significant number of CD4 cell count [3] and subsequently it leads to an increased number of opportunistic infections in PLHA. Although 67% of total PLHA were covered with antiretroviral therapy (ART) globally, the secondary infections by fungi, bacteria, parasites and other viruses have not reduced as expected. Bacterial and fungal infections are common opportunistic infections, followed by parasitic and viral infections in PLHA [4]. Similarly, 19,410 PLHA are currently receiving antiretroviral therapy in Nepal. AIDS-related deaths are declining in Nepal because of improved access to ART with 80 ART sites and 20 ART dispensing centers in 61 districts. Among the HIV-related deaths, the primary cause is advanced HIV disease (60.6%), followed by tuberculosis (22.7%). However, bacterial lower respiratory tract infections (LRTIs) other than *Mycobacterium tuberculosis* cannot be avoided in PLHA. The third important cause of death among PLHA is pneumonia (4.2%). *Klebsiella pneumoniae, Haemophillus influenzae, Streptococcus pneumoniae, Pseudomonas aeruginosa, Staphylococcus aureus, Acinetobacter* spp, *Escherichia coli* are some common bacterial pathogens causing LRTIs in PLHA [5, 6]. The development of antimicrobial resistance to commonly prescribed antibiotics among those bacterial pathogens is a matter of concern nowadays due to the increase in the ineffectiveness of antibiotic therapy in PLHA [7].

In the context of Nepal, 2000–3000 people die of AIDS-related infections every year due to a lack of effective treatment and care [2]. In most developing countries, the lack of proper diagnosis and treatment of secondary bacterial infection is deteriorating the quality of life of PLHA. However, very few studies have documented such infections and their impacts on PLHA in Nepal. Hence, this study focused on identifying potential bacterial pathogens of LRTIs among PLHA and their antibiogram phenotypically and genotypically. Moreover, we also evaluated the efficacy of co-trimoxazole, a WHO-recommended broad-spectrum antibiotic, in the treatment of pneumonia in PLHA. Finally we recommend a few potential antimicrobial agents for treating LRTIs among those patients.

## Methods

### Study design, site and duration

A hospital-based prospective cross-sectional study was conducted in Antiretroviral Treatment (ART) center of Sukraraj Tropical and Infectious Disease Hospital, one of the tertiary care hospitals with 100 beds capacity in Kathmandu, Nepal from February to August 2019. The hospital was the first to start ART service in Nepal in 2004. Around 4200–4500 PLHA visit the ART center for therapy annually. Sample collection from the participants and processing were done in the same hospital. In addition, further processing of plasmid DNA extraction and gene detection were done in the Central Department of Microbiology, Tribhuvan University, Kathmandu.

### Inclusion and exclusion criteria

All age groups of both sexes living with HIV, under ART and with LRTIs confirmed by a physician after physical examination, blood investigations including CBC and chest x-ray, were enrolled n this study. Sputum samples and the demographic information from the participants were collected only after obtaining written consent. However, the patients infected with tuberculosis were excluded from the study.

### Sample size

The calculated sample size was 255 based on the 21% prevalence rate of bacterial LRTIs in PLHA as reported by Kandati et al. [8]**.** Therefore, for this study, 263 sputum samples were collected.

### Sample collection, transportation and processing

Sputum sample were collected from the participants using sterile vials with a wide mouth and tight lid. Participants were instructed first to take a deep breath and then to expectorate a cough [9]. The collected sputum samples were transported to the Microbiology laboratory for further processing. The quality of sputum was checked macroscopically for the presence of mucopurulent part before accepting the sample.

### Sputum culture

Mucopurulent sputum was cultured on MacConkey Agar (MA), Blood Agar (BA), and Chocolate Agar (along with 10-unit bacitracin disk) and incubated at 37 °C for 24 h. Incubated plates were examined for the presence of distinct colonies, and identification was made using standard microbiological protocols, including their colony morphology on different culture media, microscopically by Gram staining and various biochemical tests [9].

For identification of *H. influenza*e, a satellitism test was performed. A loopful of suspected colonies of *H. influenzae* was mixed in about 2 ml of sterile physiological saline. Using a sterile swab stick, the bacterial suspension was inoculated on a plate of blood agar, and a pure culture of *S. aureus* was streaked across the inoculated blood agar plate. It was then incubated in a CO_2_ enriched atmosphere at 35–37 °C for 18–24 h. The culture plate was examined for growth and satellite colonies [9].

### Antibiotics susceptibility test

Out of 67 bacterial isolates from sputum culture, only 53 isolates excluding *H. influenzae* (n = 14) were processed further. Antibiotic susceptibility patterns of those organisms were performed using the modified Kirby Bauer disk diffusion method recommended by CLSI 2019 [10]. The antibiotics used were amoxicillin (10 µg), amoxicillin/clavulanic acid (AMC, 20/10 mcg), cefepime (CPM, 30mcg), ceftazidime (CTZ, 30 mcg), ceftriaxone (CTR, 30 mcg), chloramphenicol (C, 30 mcg), ciprofloxacin (CIP, 5 mcg), colistin (CL 10 mcg), co-trimoxazole (COT, 25 mcg), gentamycin (GEN, 10 mcg), imipenem (IMP, 10 mcg), piperacillin/tazobactam (PIT, 100/10 mcg), polymyxin-B (PB, 300 units) and tetracycline (TET, 30 mcg). The isolates resistant to at least one agent in ≥ 3 antimicrobial categories were considered MDR [11]. Subsequently, the prevalence of MDR bacteria was determined.

### Screening and confirmation of extended-spectrum β-lactamase (ESBL)

The isolates resistant to antibiotics—ceftazidime (30 µg) or ceftriaxone (30 µg) were considered as potential ESBL producers. The confirmatory test was performed by double disk diffusion method using ceftazidime and ceftazidime clavulanic acid. More than 5 mm zone of diameter around ceftazidime-clavulanic acid disc than ceftazidime disc alone was confirmed an ESBL producer [10].

### Detection of metallo β-lactamase (MBL)

Two imipenem discs were placed on an MHA plate inoculated with a test organism (bacterial density equivalent to 0.5 McFarland Standard). A 5 µl of EDTA (0.5 M, pH = 8.0) solution was added to one of the imipenem discs and incubated for 16–18 h at 37 °C. An increased zone of diameter (> 7 mm) around the imipenem and EDTA disc than the imipenem alone was confirmed MBL positive [10]**.**

### Bacterial plasmid DNA extraction

Plasmid DNA from MDR isolates was extracted by the alkaline lysis method followed by purification with phenol–chloroform. The isolated plasmid DNAs were visualized on 0.8% agarose gel electrophoresis with 0.2 μg/ml concentration of ethidium bromide as described by Sambrook, 1989 and Thapa Shrestha and Adhikari 2014 [12, 13].

### Molecular detection of ***bla***_CTX-M_ and ***bla***_***TEM***_ gene

A set of primers for each gene was selected from the previous studies and verified on NCBI BLAST. A set of primers (Forward: 5′-TTTGCGATGTGCAGTACCAGTAA-3′ and reverse: 5′-CTCCGCCTGCCGGTTTTAT-3′) as described in Edelsteint et al. [14] were used for amplification of *bla*_CTX-M_ gene. Similarly, a set of primers (Forward: 5′-GAGACAATAAGGGTGGTAAAT-3′ and reverse: 5′-AGAAGTAAGTTGGCAGCAGTG-3′) as mentioned in Sharma et al. 2013 were used to amplify *bla*_TEM_ gene [15]. A conventional PCR was used to amplify the *bla*_TEM_ and *bla*_CTX-M_ genes. A PCR reaction mixture was prepared containing 12.5 µl master mix (Qiagen), 0.5 µl of each primer, 3 µl template DNA and 8.5 µl PCR grade water [14]. PCR amplification was run at initial denaturation of 95 °C for 15 min; followed by 35 cycles of denaturation at 94 °C for 45 s, annealing at 55 °C for *bla*_TEM_ genes and 56 °C for the *bla*_CTX-M_ gene for 30 s, extension at 72 °C for 3 min and a final extension at 72 °C for 10 min. The PCR products were analyzed on 1.5% agarose gel electrophoresis with 0.2 μg/ml concentration of ethidium bromide and visualized under UV transilluminator.

### Quality control

Control strain of *E. coli* (ATCC 25922) and *P. aeruginosa* (ATCC 27853) were used as positive controls for ESBL and MBL producing strains, respectively. For PCR, a previously harvested plasmid DNA containing target genes were used as positive control and nuclease-free water as negative control.

### Data management and analysis

All data collected were analyzed using SPSS-21 software. The Chi-square test was used to assess the association of different variables. p-value < 0.05 was considered as significant.

## Results

**Fig. 1:**
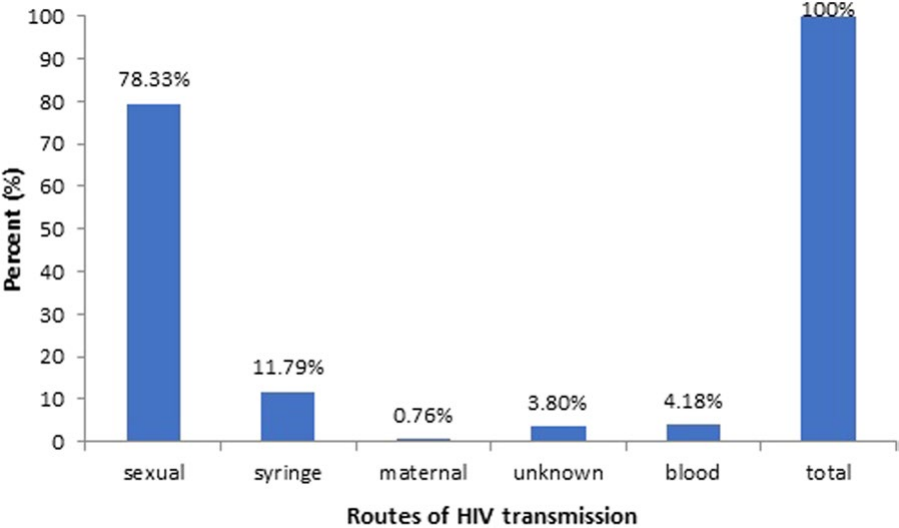
Mode of transmission of HIV among participants

*Mode of transmission of HIV among study participants:* Among 263 PLHA, 145 (55.13%) were male, and 118 (44.87%) were female. Heterosexual activities were the most dominant transmission route (78.33%), whereas the least transmission rate, 0.76% (n = 2), was observed cia a vertical route from mother to infants (Fig. 1).

*Bacterial growth according to age and gender of participants:* Overall, 67 (25.48%) sputum samples were positive for bacterial growth. The bacterial infection of LRT was found highest (50%) in the age group 61–70 years, followed by 40% (n = 10) among the males aged 51–60 years. No infection was observed among participants below 20 years (Table 1).

**Table 1:** Age and sex-wise distribution of Microbial growth

Age group (years)	Male		Female		Total number (growth %)
	No. of sample	Growth (%)	No. of sample	Growth (%)	
Below 20	3	0	1	0	4 (0)
21–30	29	8 (27.59)	20	5 (25)	49 (26.53)
31–40	40	9 (22.5)	52	12 (23.08)	92 (22.83)
41–50	42	10 (23.81)	32	10 (31.25)	74 (27.03)
51–60	25	10 (40)	11	1 (9.09)	36 (30.56)
61–70	6	3 (50)	2	1 (50)	8 (50)
Total	145	40 (27.59)	118	27 (22.88)	263 (25.48)

*Bacterial co-infection and predisposing factors:* Bacterial infection was significantly higher (p = 0.02) among particiapnts with CD4 count < 200 cells/µl. Similarly, bacterial infections were higher among smokers and those with a history of pulmonary tuberculosis, but the data was not statistically significant (Table 2).

**Table 2:** Relation bacterial infection with predisposing factors

Predisposing factors	Status of predisposing factors	Bacterial Growth		Total	p-value
		Negative (%)	Positive (%)		
CD4 count cells/µl	< 200	10 (47.62)	11 (52.38)	21	0.02
	200–499	56 (60.22)	37 (39.78)	93	
	> 500	130 (87.25)	19 (12.75)	149	
Total		196 (74.52)	67 (25.48)	263	
Smoking habit	Non-smoker	117 (77.48)	34 (22.52)	151	> 0.05
	Smoker	35 (70)	15 (30.0)	50	
	Previous smoker	44 (70.97)	18 (29.03)	62	
Total		196 (74.52)	67 (25.48)	263	
History of PTB	No cases	149 (77.20)	44 (22.80)	193	> 0.05
	Cases	47 (67.14)	23 (32.86)	70	
Total		196 (74.52)	67 (25.48)	263	

*Bacterial infection based on their literacy and occupation:* Most samples (43.3%) were from PLHA who had completed their primary level education. The bacterial infection was found higher among literate people as compared to illiterate PLHA. Likewise, bacterial infection was higher (30%) among business people followed by office workers (26.6%) (Table 3).

**Table 3:** Microbial growth pattern and literacy

Literacy	Number of sample	Bacterial growth (%)	Occupation	Number of sample	Bacterial growth (%)
Illiterate	85	26 (30.59)	Households	82	20 (24.39)
Primary	114	28 (24.56)	Farmer	36	10 (27.78)
Secondary	52	8 (15.38)	Business	50	15 (30)
Higher secondary	9	3 (33.33)	Driving	13	2 (15.38)
Bachelors	3	2 (66.67)	Social work	10	2 (20)
Total	263	67 (25.48)	Labor	9	2 (22.22)
			Official work	38	10 (26.32)
			Others	25	6 (24)
			Total	263	67 (25.48)

**Fig. 2:**
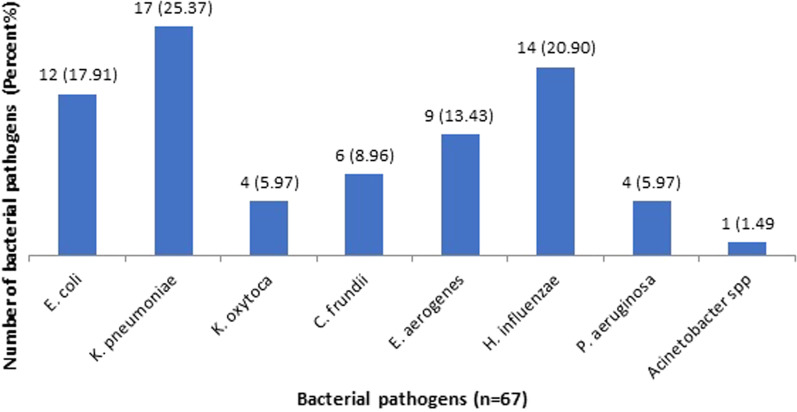
Distribution of bacterial pathogens in LRT infections of HIV people

*Frequency of bacterial pathogens from LRT infections:* Of 67 Gram negative bacterial isolates, *K. pneumoniae* was the most predominant bacteria followed by *H. influenzae* (Fig. 2).

*Antibiotic susceptibility pattern:* All isolated strains of *K. pneumoniae* and *K. oxytoca* were resistant to amoxicillin, whereas all of them were sensitive to colistin and polymyxin B. Of 12 isolates of *E. coli*, 10 (83.33%) were resistant to amoxicillin, and 9 (75%) were resistant to third-generation cephalosporins. Similarly, nearly 90% of *E. aerogenes* (8/9) were resistant to amoxicillin. All isolates of *C. frundii* were resistant to amoxicillin (Table 4).

**Table 4:** Antibiogram of bacterial isolates of *Enterobacteriaceae* family

Antibiotics used	Number of resistant pathogens (%)				
	*K. pneumoniae* (n = 17)	*K. oxytoca* (n = 4)	*E. coli* (n = 12)	*E. aerogenes* (n = 9)	*C. frundii* (n = 6)
Amoxicillin	17 (100)	4 (100)	10 (83.33)	8 (88.89)	6 (100)
Cotrimoxazole	10 (58.82)	2 (50)	8 (66.67)	1 (11.11)	0
Ceftriaxone	9 (52.94)	2 (50)	9 (75)	1 (11.11)	0
Ciprofloxacin	4 (23.53)	1 (25)	7 (58.33)	1 (11.11)	0
Chloramphenicol	3 (17.65)	0	3 (25)	1 (11.11)	0
Gentamycin	2 (11.76)	0	1 (8.33)	1 (11.11)	0
Tetracycline	14 (82.35)	3 (75)	7 (58.33)	3 (33.33)	3 (50)
Ceftazidime	9 (52.94)	2 (50)	9 (75)	1 (11.11)	0
Amoxicillin-clavulanic acid	10 (58.82)	2 (50)	9 (75)	2 (22.22)	0
Piperacillin tazobactam	5 (29.41)	1 (25)	7 (58.33)	1 (11.11)	0
Cefepime	5 (29.41)	1 (25)	5 (41.67)	1 (11.11)	0
Imipenem	2 (11.76)	0	1 (8.33)	1 (11.11)	0
Polymyxin B	0	0	0	0	0
Colistin	0	0	0	0	0

Besides the intrinsic resistance of *P. aeruginosa* to many antibiotics, including amoxicillin, co-trimoxazole, ceftriaxone, ceftazidime, chloramphenicol, tetracycline, amoxicillin-clavulanic acid, piperacillin-tazobactam and 3 strains (75%) were also resistant to ofloxacin and cefepime. However, all strains were sensitive to gentamycin, ciprofloxacin, imipenem, amikacin, and colistin. Likewise, *Acinetobacter* spp were sensitive to gentamycin, imipenem, polymyxin B and colistin out of 14 antimicrobials used in our study.

*Multidrug-resistant (MDR) and extended-spectrum β-lactamase (ESBL) producing strains:* Out of 53 bacterial isolates, 28 (52.83%) were multidrug-resistant (MDR). All strains of *P. aeruginosa* and *Acinetobacter* spp were found to be MDR strains whereas no strain of *C. frundii* were MDR. Similarly, 75% (9/12) of *E. coli,* 64.71% (11/17) of *K. pneumoniae*, and 50% (2/4) of *K. oxytoca* were MDR strains.

A total of 23 (43.4%) isolates were ESBL producers, including 75% (9/12) *E. coli* and all isolates of *P. aeruginosa* and *Acinetobacter* spp. Of the total MDR strains, 72.73% (8/11) of *K. pneumoniae* and 50% (1/2) of *K. oxytoca* were ESBL producers. In addition, one strain of *P. aeruginosa* was found MBL producer.

**Fig. 3:**
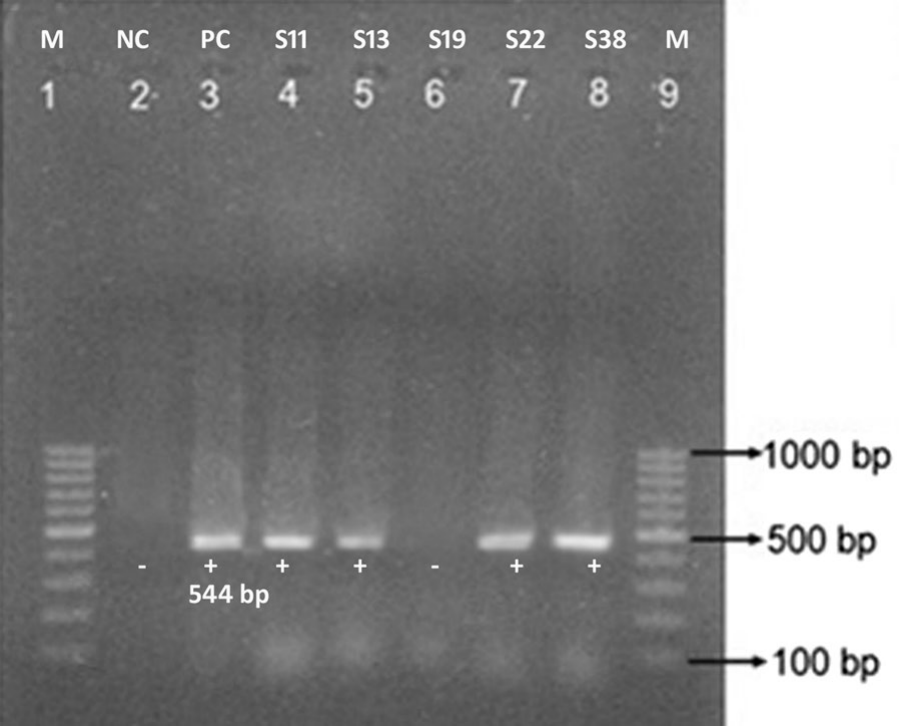
Amplification of *bla*_*CTM*_ gene (Lane 1 and 9; DNA marker (100 bp), Lane 2; NC (Negative control), Lane 3; PC (Positive control) and Lane 4–8; gene amplification from isolates) (The photo has been cropped to clearly show the bands of *bla*_*CTM*_ gene, the original photo can be submitted as supplementary on request)

**Fig. 4:**
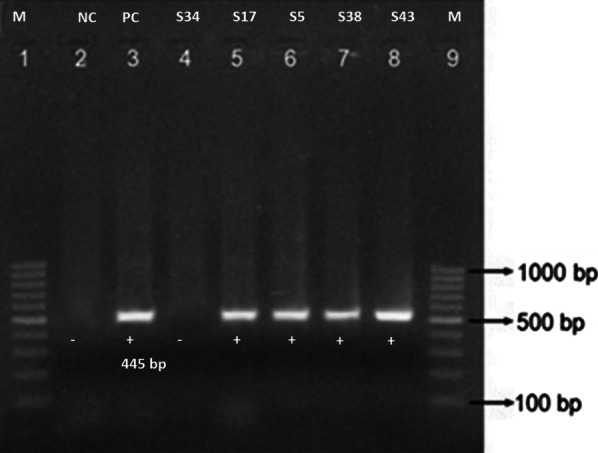
Amplification of *bla*_*TEM*_ gene (Lane 1 and 9; DNA marker (100 bp), Lane 2; NC (Negative control), Lane 3; PC (Positive control) and Lane 4–8; gene amplification from isolates) (The photo has been cropped to clearly show the bands of *bla*_*TEM*_ gene, the original photo can be submitted as supplementary on request)

*Detection of ESBL genes:* Of 53 isolates, 23 (43.4%) possessed ESBL genes. Among them, 11 (47.83%) harbored *the bla*_CTX-M_ gene (Fig. 3) and 2 (8.69%) contained *the bla*_TEM_ gene (Fig. 4). Similarly, ten isolates (43.48%) possessed both *bla*_TEM_ and *bla*_CTX-M_ genes in their plasmid DNA (Table 5).

**Table 5:** Detection of *bla*_CTX-M_ and *bla*_TEM_ ESBL genes among the isolates

Isolates	n	Phenotypic ESBL	Genotypic ESBL	Number of amplified genes		
				*bla*_CTX-M_ (%)	*bla*_TEM_ (%)	*bla*_CTX-M_ + *bla*_TEM_ (%)
*K. pneumoniae*	17	8 (47.06)	7 (41.18)	3 (42.86)	0	4 (57.14)
*K. oxytoca*	4	1 (25)	2 (50)	1 (50)	0	1 (50)
*E. coli*	12	9 (75)	9 (75)	3 (33.33)	2 (22.22)	4 (44.45)
*P. aeruginosa*	4	4 (100)	3 (75)	3 (100)	0	0
*Acinetobacter* spp	1	1 (100)	1 (100)	1 (100)	0	0
*E. aerogenes*	9	0	1 (11.11)	0	0	1 (100)
*C. frundii*	6	0	0	0	0	0
Total	53	23 (43.4)	23 (43.4)	11 (47.83)	2 (8.69)	10 (43.48)

## Discussion

Among the different routes of HIV transmission, most participants in this study were infected through the heterosexual route (78.33%). Similar to this study, Chandwani et al. reported the highest transmission rate (95%) via the heterosexual route [16]. Almost all female participants in our study were housewives and got HIV infections from their husbands. The least rate of transmission from mother to infants might be due to increased awareness on HIV-AIDS and wide coverage by ART, reducing the rate of transmission as recommended by WHO [1].

Of 263 samples processed, 67 (25.5%) were found culture positive. Similarly, KC et al. and Ojha et al. reported 24% and 46.6% of bacterial infections among PLHA, respectively, from tertiary care hospitals in Kathmandu [5, 6]. KC et al. reported a higher rate of fungal infection (42%) as compared to bacterial infection (24%) in the same hospital from August 2017 to March 2018 [6]. Likewise, Oja-Bola and Oluyege reported 55.6% of PLHA to be associated with pneumonia [7]. The comparatively lower rate of bacterial infections might be due to the co-trimoxazole prophylaxis which is recommended for people under ART by WHO [17]. In addition, PLHA having lower CD4 cells were given Isoniazid preventive treatment to prevent MTB [18, 19]. A relatively higher occurrence of LRTIs was observed in the old aged 61–70 years as compared to other age groups (p-value 0.49). The weakness of the immune system with age makes PLHA vulnerable to different types of infections. Macfarlane et al. 1993 also reported the same [20]. No infection was reported in young participants of aged below 20. The infection rate was higher in male participants (27.5%) than female participants (22.88%). Similar to our study, Ojha et al. reported the rate of infection in males and females in the ratio of 1.3:1 (p = 0.39) [5].

Many predisposing factors are associated with bacterial co-infection in PLHA. A significantly higher rate of bacterial infection (39.8%) was observed in participants having CD4 count < 200 cells/µl as compared to the least infection in those with CD4 count > 500 cells/µl. Yadav and Prakash also observed a significantly higher rate of LRTIs (63.4%) among cases with a CD4 count < 200 cells/µl, followed by those within the 200–500 range category (53.1%) and least (18.7%) in that above 500 cells/µl [21]. The most important risk factor for bacterial pneumonia in PLHA is the degree of immunosuppression reflected by the CD4 + T-lymphocyte count [22]. On the other hand, a slightly higher bacterial infection rate was observed in active smokers (p = 0.43). In contrast, Yadav and Prakash reported a significantly higher rate of LRTIs among smokers [21]. They also identifed tobacco smoking as one of the most important risk factors contributing to Nepal’s higher prevalence of chronic bronchitis and chronic obstructive lung disease. Various mechanisms cause increasing susceptibility of smokers to different infections, including structural changes in the respiratory tract and decreased immune response [23]. Thirdly, bacterial infection in PLHA previously infected with pulmonary tuberculosis (PTB) were comparatively higher than those without a history of PTB (22.8%) (p = 0.06). Since PTB is a chronic lung disease and leaves patches, pulmonary nodules, or granuloma in the lungs, it might enhance the secondary bacterial infection [24]. Likewise, people who are more exposed to the external environment such as shopkeepers, carpenters, tourist guides, security guards, army, police, etc., were found more susceptible to LRTIs than other people involved in household activities. The reason might be due to exposure to the polluted outdoor environment.

Among 67 Gram-negative isolates, *K. pneumoniae* was the predominant one, followed by *H. influenzae* and *E. coli*. Similar to this study, Jemal et al. found *K. pneumoniae* as a predominant one, accounting for 41.3% [25]. Few other studies have also reported *K. pneumoniae* as the predominant bacteria causing LRTIs among PLHA [5, 6]. In contrast, Oja-Bola and Oluyege reported *E. coli* (40%) as the most frequent organism, followed by *P. aeruginosa* (35%) and *K. pneumoniae* as the least (5%) isolated one [7]. *K. pneumoniae* can cause diseases in non-HIV people as well; however, pneumonia due to *Klebsiella* is classically thought to be community-acquired [6]. However, Mishra and the coworkers reported *H. influenzae* as the most predominant (21%) isolate in the sputum of PLHA [26]. *H. influenzae* associated pneumonia is highly associated with a high degree of immune suppression [27].

We found amoxicillin, tetracycline, and co-trimoxazole to be the least effective drugs against Gram-negative pathogens; however, WHO has recommended co-trimoxazole to treat pneumococcal disease in PLHA. This result was also supported by Oja-Bola and Oluyege [7]. They found only 20% of *Klebsiella* spp were sensitive towards co-trimoxazole. Similarly, Adeleye et al. reported co-trimoxazole to be resistant for LRT isolates in Nigeria [28]. The resistance towards trimethoprim-sulfamethoxaxole might be due to the extensive use of co-trimoxazole by PLHA as their basic regimen for treating opportunistic bacterial infections. In the study, ESBL producing strains were resistant towards most of the antibiotics used except gentamycin, imipenem, polymyxin B and colistin, while non-ESBLproducing isolates were sensitive towards all antibiotics except amoxicillin and tetracycline.

Plasmid-mediated ESBL producers are nowadays a matter of concern due to their capacity to hydrolyze 3rd and 4th generation cephalosporin and monobactams [29]. Decreased susceptibility of Gram-negative isolates towards 3^rd^ and 4^th^ generation cephalosporin could be attributed to ESBL or AMP-C beta-lactamase production. The most predominant ESBL producers were found to be *E. coli, P. aeruginosa* and *Acinetobacter* spp, followed by *K*. *pneumoniae* by the phenotypic method*.* Contrary to our result, KC et al. reported *K. pneumoniae* (80%) and *P. aeruginosa* (50%) as the two predominant species to produce ESBL from the same study site [6].

On amplifying the plasmid-mediated ESBL gene, 23 (43.3%) isolates gave a positive result. The two isolates that gave positive ESBL test phenotypically contained neither of the two genes. It might be due to the presence of ESBL genes other than targeted ones. This result is supported by those from de Oliveira et al. [30]. Another two isolates possessed ESBL genes but were phenotypically undetectable by combined disk test, highlighting the sensitivity of the genotypic method over the phenotypic method. Another reason might be a low level of ESBL gene expression. Gautam et al. also reported 40.8% of PCR-positive ESBL producers were phenotypically undetectable [31]. They also explained that phenotypic identification of ESBL was based on the inhibition of the enzyme by clavulanic acid, and the inhibitory action of clavulanic acid could be masked due to the co-existence of multiple enzymes. In addition, the co-existence of AmpC enzymes in ESBL producers may alter the pores of the cell membranes, resulting in reduced affinity for β-lactamase inhibitors for enzymes such as TEM and SHV. Hence, the production of different types of β-lactamases (TEM, SHV, CTX-M and OXA) by the same microorganism can lead to erroneous phenotypic conclusions.

As a significant study limitation, we couldn’t analyze the antibiotic susceptibility pattern of *H. influenzae* due to the lack of X and V factors in the hospital laboratory at the time of research. In addition, all possible ESBL genes were not detected because of the low budget and limited timeframe for the study. Instead, we observed a few antimicrobial agents such as gentamycin, imipenem, polymyxin B and colistin*,* that have higher effectiveness to inhibit all MDR and ESBL producing bacterial strains. These antimicrobial agents can be a choice of drugs for treating LRTIs in PLHA.

## Conclusion

LRTIs other than PTB by MDR Gram-negative pathogens are common opportunistic infections deteriorating the quality of life of PLHA. Furthermore, the higher rate of resistance towards the WHO-recommended broad-spectrum antibiotic—co-trimoxazole and the presence of drug-resistant genes call for an alarm against the rampant use of antibiotics among PLHA.

## Data Availability

The datasets used and/or analyzed during the current study are available from the corresponding author on reasonable request at upendrats@gmail.com.

## Abbreviations

AIDS: Acquired immune deficiency syndrome
ATCC: American type culture collection
ART: Antiretroviral treatment
AST: Antibiotic susceptibility testing
BA: Blood agar
CA: Chocolate agar
CDC: Center for diseases control and prevention
CLSI: Clinical Laboratory Standard Institute
DOHS: Department of health service
ESBL: Extended spectrum β-lactamase
HIV: Human immunodeficiency virus
LRTI: Lower respiratory tract infection
MA: Mac-Conkey agar
MBL: Metallo β-lactamase
MHA: Mueller hinton agar
DR: Multidrug resistance
min: Minute/s
PCR: Polymerase chain reaction
PLHA: People living with HIV-AIDS
PTB: Pulmonary tuberculosis
STIDHH: Sukraraj Tropical and Infectious Disease Hospital
WHO: World Health Organization
